# Plumbagin Is a PKM2 Activator That Modulates Glutamine Metabolism and Dependency in Leukemia

**DOI:** 10.3390/metabo16080577

**Published:** 2026-08-16

**Authors:** Nikolina Vrdoljak, Mark D. Minden, Paul A. Spagnuolo

**Affiliations:** 1 Department of Food Science, University of Guelph, Guelph, ON N1G 2W1, Canada; nvrdolja@uoguelph.ca; 2 Princess Margaret Cancer Centre, Ontario Cancer Institute, Toronto, ON M5G 2M9, Canada; mark.minden@uhn.ca

**Keywords:** acute myeloid leukemia (AML), metabolism, pyruvate kinase M2 (PKM2), metabolic reprogramming, nutraceutical, glutamine metabolism, glutaminase-1 (GLS-1)

## Abstract

Background: AML cells can be defined by impairments in glycolytic metabolism, resulting in increased glucose uptake coupled with reduced glycolytic flux. Consequently, cells rely on alternative pathways such as glutamine metabolism to fuel mitochondrial respiration through anapleurosis. AML cells express upregulated levels of glutamine transporters and catabolic enzymes such as solute carrier family 1 member 5 (SLC1A5) and glutaminase 1 (GLS-1), respectively, to support metabolic needs; impairment of glutamine metabolism induces proliferative arrest. Our previous work identified plumbagin (PLB) as a selective activator of pyruvate kinase isoform M2 (PKM2), resulting in increased PKM2 tetrameric protein, impaired PKM2 nuclear translocation and suppressed c-Myc expression. Objective: Therefore, we aimed to investigate whether PLB-mediated PKM2 activation influences glutamine metabolism as a downstream effect of c-Myc suppression in AML. Methods/Results: AML cell lines treated with PLB were cultured in the presence or absence of glutamine and were compared to cell models with genetically suppressed PKM2 to assess for differences in growth. Spectrophotometric analysis revealed that PLB treatment reduces intracellular glutamine uptake, and immunoblotting indicated suppression of GLS-1 expression, ultimately leading to reduced AML cell proliferation and viability. Supplementation with glutamine partially restored cell growth, indicating that PKM2 modulation is associated with impaired glutamine uptake and utilization. Conclusion: Overall, this study explores the downstream implications of PLB-induced alterations in the c-Myc/PKM2 axis, expanding the understanding of PKM2’s function beyond glycolysis. The findings presented confirm that PKM2 activation leads to indirect consequences on glutamine metabolism in AML, providing further insight into the mechanisms of PLB-mediated AML cell death.

## 1. Introduction

**Figure 1:**
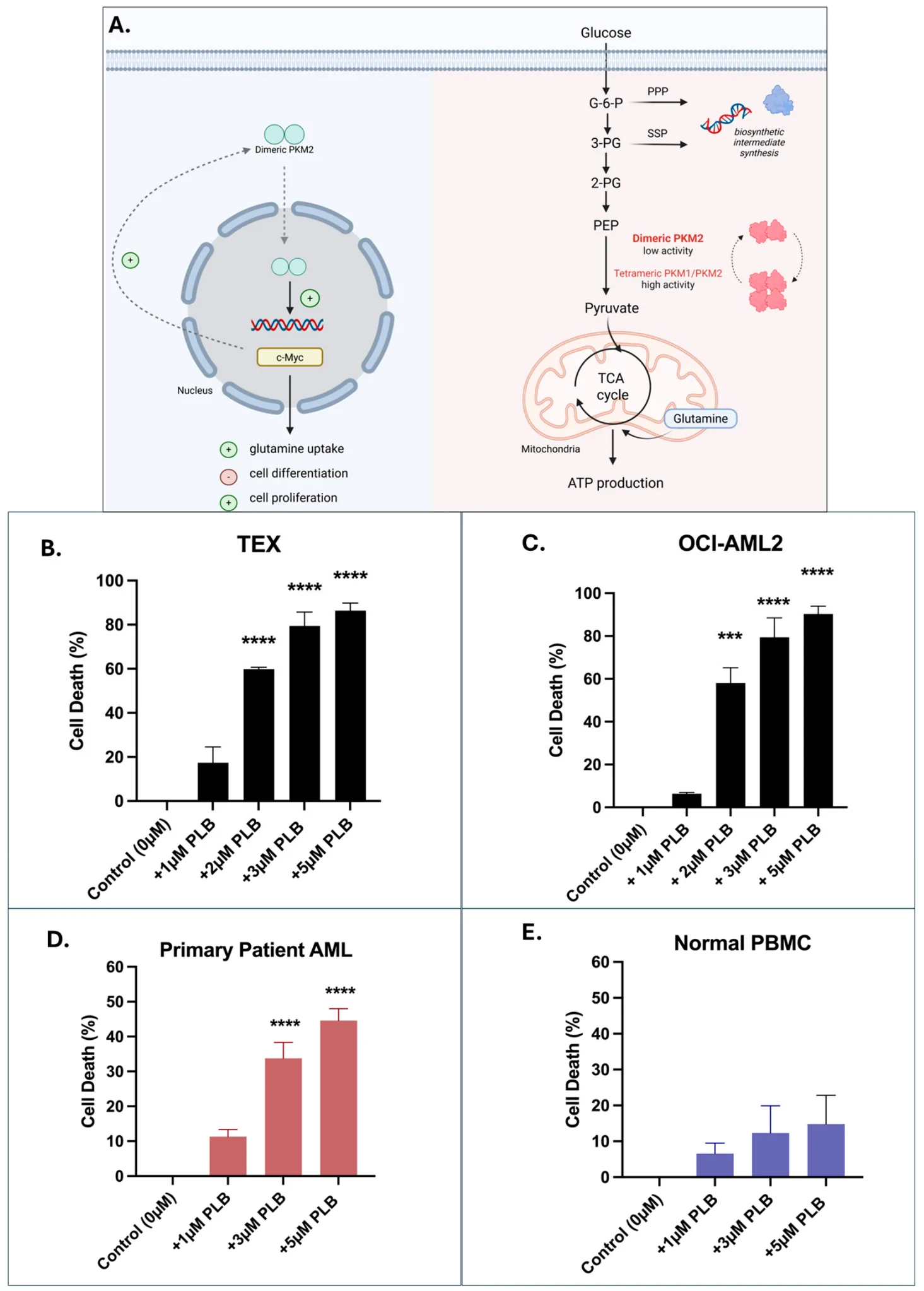
PLB affects the protein and pyruvate kinase roles of PKM2 in AML. (**A**) Schematic diagram of the functional roles of pyruvate kinase isoform M2 (PKM2). (**Left panel**): Dimeric PKM2 is imported into the nucleus and acts as a transcriptional co-activator, increasing the expression of c-Myc, leading to increased glutamine uptake and cell proliferation while inhibiting myeloid cell differentiation. c-Myc increases heterogenous nuclear ribonucleoproteins (hnRNPs) that promote the splicing of the PKM2 isoform. (**Right panel**): The metabolic role of pyruvate kinase (PK) in the cytosol. Dimeric PKM2 possesses lower enzymatic activity, leading to an accumulation of upstream glycolytic intermediates that are diverted to anabolic pathways. Alternative fuel sources including glutamine are used to fuel the tricarboxylic acid (TCA) cycle. Biorender, + N. Vrdoljak (2025) + http://Biorender.com. (**B**) Cell death was analyzed following 72 h of treatment in TEX and (**C**) OCI-AML2 cell lines, as well as (**D**) primary acute myeloid leukemia (AML) patient samples (ID #1-8) and (**E**) peripheral blood mononuclear cells (PBMCs) from healthy donors (n = 3). Cytogenetics for patient-derived AML samples are provided in Table 1. *** *p* ≤ 0.001; **** *p* ≤ 0.0001. One-way ANOVA with Tukey’s post hoc test. All data is represented as the mean ± standard error of mean. G-6-P: glucose-6-phsophate; 3-PG: 3-phosphoglycerate; 2-PG: 2-phosphoglycerate; PEP: phosphoenolpyruvate; PPP: pentose phosphate pathway; SSP: serine synthesis pathway; ATP: adenosine triphosphate. (**B**,**C**), n = 3 biological replicates.

Acute myeloid leukemia (AML) is an aggressive malignancy of the blood and bone marrow, with a dismal prognosis and sub-optimal therapeutics [1]. Pharmacological targeting of AML cell metabolism, characterized by a shift towards aerobic glycolysis (AG) and away from oxidative phosphorylation (OXPHOS), has emerged as an attractive therapeutic approach [2,3,4,5,6,7]. Instead of utilizing glucose-derived pyruvate for mitochondrial energy respiration, an increased proportion of glucose-derived carbons are diverted to biosynthetic pathways critical for promoting cell growth (i.e., pentose phosphate pathway and serine synthesis pathway) [5,8,9,10]. As a result, compensatory pathways such as glutaminolysis are employed to sustain mitochondrial ATP production (Figure 1A, right panel) [5,11]. Malignant cells become highly reliant on glutamine, the most abundant non-essential amino acid in the body [12]. In AML patients, blood glutamine concentrations are reduced by approximately 50%, highlighting fast consumption and utilization [13].

Given the dependence on glutamine metabolism, pharmacological inhibition of this pathway has emerged as a potential therapeutic strategy. Therapeutic approaches include inhibiting glutamine uptake through targeting glutamine transporter solute carrier family 1 member 5 (SLC1A5), as well as pharmacological inhibition of glutaminase 1 (GLS-1), a catabolic enzyme responsible for initiating glutaminolysis [14,15,16]. These proteins are upregulated in various malignancies, including leukemia [14]. c-Myc is an oncogenic transcription factor that upregulates the transcription of these transporters (Figure 1A, left panel) and promotes the expression of catabolic enzymes associated with glutaminolysis (i.e., GLS-1) [17,18,19]. c-Myc expression itself is regulated through various mechanisms, including a positive feedback loop involving the M2 isoform of pyruvate kinase (pyruvate kinase isoform M2; PKM2) [20,21]. This isoform of the glycolytic enzyme is overexpressed in malignancies including AML; PKM2’s dimeric conformation is associated with reduced enzyme activity and pyruvate production compared to its counterpart, the M1 isoform (PKM1). The PKM1 isoform is normally expressed as a constitutively active tetramer in several healthy tissue types [22,23,24]. Interestingly, aside from its pyruvate kinase role, dimeric PKM2 can translocate to the nucleus to act as a protein kinase and transcriptional co-activator, enhancing c-Myc transcription and affecting downstream signaling pathways [25,26,27].

Modulation of PKM2 has therefore been investigated as an anti-cancer strategy [23,28,29,30]. Several pharmacological activators of PKM2 have been reported, including synthetic compounds such as ML-265 and DASA-58, which promote PKM2 tetramerization and alter glycolytic metabolism by increasing pyruvate kinase activity [23]. Micheliolide is a natural compound classified as a sesquiterpene lactone, with half-maximal inhibitory concentration (IC_50_) values ranging from 3.9 to 16.2 µM in a panel of cell lines including AML cell lines KG1a and HL-60. Micheliolide irreversibly and specifically activates PKM2, increasing tetramer formation and reducing nuclear PKM2 accumulation [30]. In pre-clinical models, ML-265 and micheliolide suppress tumor growth, validating PKM2 activation as a viable treatment strategy [31,32].

Recently, plumbagin (PLB) was identified as a direct pharmacological activator of PKM2 [33,34]. PLB is a naturally occurring naphthoquinone compound derived from the *Plumbago zeylanica* plant that has gained attention due to its anti-cancer properties. PLB has been shown to induce cell death in numerous solid and hematological tumor types through modulation of cell signaling pathways and redox reactions, such as inhibition of STAT3 phosphorylation resulting in impaired cancer cell metabolism [35,36,37,38,39,40,41]. We previously demonstrated that PLB binds to and selectively activates PKM2, increasing tetrameric conformation, reducing nuclear translocation and suppressing expression of c-Myc, ultimately resulting in selective AML cell death [34]. We reported IC_50_ values of 1.89 µM in TEX cells and 1.98 µM in OCI-AML2 cell lines, highlighting potent cytotoxicity. In addition, pre-clinical studies demonstrate that following intraperitoneal injection of PLB at a dose of 2.5 mg/kg, three times a week, in vivo engraftment of AML is reduced by approximately 85% [34]. This provides a framework by which reported downstream pathways, including STAT3 inhibition, may be understood.

Identifying PKM2 as the molecular target of PLB and characterizing its effects on nuclear localization and c-Myc expression allow for investigation of the downstream metabolic consequences of PKM2 activation. Given the multifaceted roles of PKM2 in regulating cell metabolism and cell signaling, further investigation of whether PLB-mediated PKM2 activation influences metabolic pathways beyond those previously characterized would provide a more comprehensive understanding of its mechanism. Building on the well-established role of c-Myc-regulated glutamine metabolism, this study demonstrates that PLB-induced PKM2 activation and subsequent c-Myc impairment result in reduced glutamine intake and metabolism in AML cells. This further supports the promising anti-AML approach of PKM2 activation, and broadens the characterization of PLB’s specific activity and therapeutic potential.

## 2. Materials and Methods

### 2.1. Cell Culture and Drugs

TEX cells were cultured in Iscove’s Modified Dulbecco’s Medium (IMDM; Cytvia, Marlborough, MA, USA) supplemented with 15% fetal bovine serum (FBS; Sigma-Aldrich, St. Louis, MO, USA), 20 ng/mL stem cell factor (Peprotech, Rocky Hill, NJ, USA), 2 ng/mL interleukin 3 (Peptrotech), 2 mM L-glutamine (Cytvia), 20 units of penicillin and 200 µg of streptomycin (per mL of media; Cytvia). The AML cell line Ontario Cancer Institute-acute myeloid leukemia-2 (OCI-AML2) cells were cultured in IMDM supplemented with 10% fetal bovine serum (FBS; Cytvia), 20 units of penicillin and 200 µg of streptomycin (per mL of media; Cytvia). Both cell lines were cultured in conditions of 37 °C, 95% relative humidity and 5% CO_2_. For glutamine depletion studies, IMDM without glutamine (Sigma-Aldrich) was prepared in the same manner. Cell lines were provided by Drs. Mark Minden and John Dick.

Primary AML samples from the peripheral blood of consenting patients, as well as peripheral blood mononuclear cells (PBMCs) from healthy donors, were obtained from the Princess Margaret Cancer Centre, with primary cells containing >80% malignant cells at the time of collection. AML patient cytogenetics are provided in Table 1. Primary cells were maintained in MethoCult H5100 media (Stem Cell Technologies, Vancouver, Canada) with 20% FBS. Additional healthy donor samples were purchased from Stem Cell Technologies (CAT# 70025.1). PLB is a naphthoquinone derived from the genus *Plumbago* and was purchased from Cayman Chemical (#14314) prior to dissolving in dimethyl sulfoxide (DMSO). PLB stocks were diluted with phosphate-buffered saline (PBS).

**Table 1:** Primary AML patient cytogenetics.

Patient ID	Cytogenetics *
AML 1	unsuccessful
AML 2	46,XX [20]
AML 3	46,XY [20]
AML 4	46,XY [20]
AML 5	46,XY [20]
AML 6	46,XY [21]
AML 7	46,XY
AML 8	46,XY [20]

* Reporting Style: overall number of chromosomes identified; sex chromosomes; in bracket, number of cells with given karyotype.

### 2.2. Cell Growth and Viability

Prior to immunoblotting, cells were cultured in the presence or absence of PLB, and total cell lysates were prepared as previously described [2,3,34]. Cells were collected and centrifuged at 2000 rpm for 10 min, supernatant was discarded, and the pellet was washed with PBS. Following this, the pellet was resuspended in radioimmunoprecipitation assay (RIPA; Sigma-Aldrich) buffer and 1× protease inhibitor cocktail (PIC; Sigma-Aldrich). Samples were then incubated on ice and vortexed periodically for 20 min, followed by centrifugation at 13,200 rpm at 4 °C for 20 min. The supernatant was isolated, and protein concentration was analyzed using a bicinchoninic acid (BCA) assay (BCA and copper II sulfate; Sigma-Aldrich). In total, 20 µg of cell lysate was diluted in 4× sodium dodecyl sulfate (SDS) sample buffer and heated at 95 °C for denaturation for 5 min. A total of 30 µL of solution was loaded onto a 10% SDS–polyacrylamide gel electrophoresis (PAGE) system and electrophoresed at 150 V for 60 min for protein separation according to molecular weight. Following this was a semi-dry transfer to a polyvinylidene difluoride (PVDF) membrane, and the membrane was then blocked in a 5% skim milk solution in 1× Tris-buffered saline with Tween20 (TBS-T) for 1 h. Membranes were incubated with primary antibody overnight at 4 °C, with antibody information detailed in Table 2. The membrane was then washed with TBS-T and incubated in secondary goat anti-rabbit IgG H&L (horseradish peroxidase (HRP); Abcam #97051, Cambridge, UK) for 1 h before washing again with TBS-T. Membranes were submerged in enhanced chemiluminescence (ECL) solution (Biorad, Hercules, CA, USA) and protein visualization was conducted using an Azure 280 Western Blot Imaging System. Image J was used to conduct densitometric analysis.

### 2.3. Lentiviral-Mediated Gene Knockdown

Lentiviral-mediated knockdown of PKM2 was conducted as previously described [2,3,34,42]. A hairpin–pLKO.1 vector containing both a puromycin-resistant gene and short hairpin ribonucleic acid (shRNA) sequences specific for PKM2 was used, and the mature antisense sequences (5′–3′) were as follows: shRNA-21, ATCTTGATGTTCTTTCCCTTC (Horizon Discovery Clone ID TRCN0000037610) and shRNA-23, TTTAGTGTCTAGAGCCACAGC (Horizon Discovery Clone ID TRCN0000037612). These shRNA constructs were sourced from the TRC-Hs1.0 (Human) library developed by The RNAi Consortium. In addition, a control with wildtype PKM2 expression containing a non-targeting shRNA (pLKO.1 backbone, Horizon Discovery) was used as is denoted as TRC.

For preparation of lentivirus, bacterial glycerol stocks of each shRNA vector along with psPAX packing and psMD2.G envelope vectors (Horizon Discovery, Cambridge, UK) were first expanded in agar. Single colonies were isolated and again expanded in agar prior to plasmid DNA extraction with a midi-prep kit (Qiagen, Hilden, Germany). A lentiviral plasmid along with psPAX2 and psMD2.G was used to transfect HEK-293T cells in the presence of TransIT (Mirus, Madison, WI, USA) transfection reagent for 24 h. Upon collection of the virus, TEX and OCI-AML2 cell lines were infected (1.4 × 10^6^ cells in 1 mL volume) with the lentivirus and protamine sulfate (20 µg/mL; Sigma-Aldrich) for 24 h. The next day, cells were collected and resuspended in IMDM containing 1 µg/mL of puromycin; every 72 h, puromycin was replaced to maintain selective pressure for one week. Knockdown was confirmed via immunoblotting, as was wildtype PKM2 expression of control (i.e., TRC) cells; all knockdown experiments are normalized to TRC cells.

### 2.4. Glutamine Uptake Assay

Glutamine uptake was measured through analysis of intracellular glutamine using a commercially available kit (Sigma-Aldrich; MAK438). According to manufacturer instructions, 20 × 10^6^ cells were collected through centrifugation at 1000× *g* for 10 min at 4 °C, followed by a washing step in cold PBS. Pellets were then resuspended in 1 mL of lysis buffer (50 mM 2-(N-morpholino)ethanesulfonic acid (MES) pH 7, 1 mM ethylenediaminetetraacetic acid (EDTA), 1 mM NaCl) followed by sonication. Samples were then centrifuged at 10,000× *g* for 15 min at 4 °C, and 20 µL of supernatant was transferred to a 96-well plate. Spectrophotometric analysis was used to quantify glutamine according to the manufacturer instructions.

### 2.5. Statistical Analysis

Statistics were analyzed using GraphPad 10.0 Prism, and results presented are the mean ± standard error of mean. The significance of the difference in values between groups was determined using an unpaired, two-tailed Student’s *t*-test or one-way analysis of variance (ANOVA) paired with Tukey’s post hoc test. *p* < 0.05 was defined as statistically significant.

No formal sample size calculation was performed, as the number of primary AML samples analyzed was determined by availability of patient samples during the period of study. Similarly, due to the small sample size of the primary AML cohort, formal tests of normality were not performed. Blinding was not feasible for Western blot quantifications as lane identities were known to the investigators; however, quantification was conducted uniformly across all samples.

## 3. Results

### 3.1. Plumbagin Imparts Selective AML Cell Death

PLB, a PKM2 activator, induced a dose-dependent increase in cell death (Figure 1B, F(4, 10) = 58.54, *p* ≤ 0.0001; Figure 1C, F(4, 9) = 89.29, *p* ≤ 0.0001). Specificity to AML was confirmed using patient-derived AML and peripheral blood mononuclear cells (PBMCs) from healthy donors. As expected, a dose-dependent increase in AML cell death was observed with no significant effect on healthy PBMCs, demonstrating that PLB is selectively toxic towards AML cells (Figure 1D,E, F(3,28) = 45.07, *p* ≤ 0.0001). Cytogenetics for patient-derived AML samples are provided in Table 1. Although the clinical feasibility of PLB is not yet established, its potency and selectivity support future work regarding translational investigation.

### 3.2. Plumbagin Reduces AML Cell Proliferation and Reduces Glutamine Dependency

**Figure 2:**
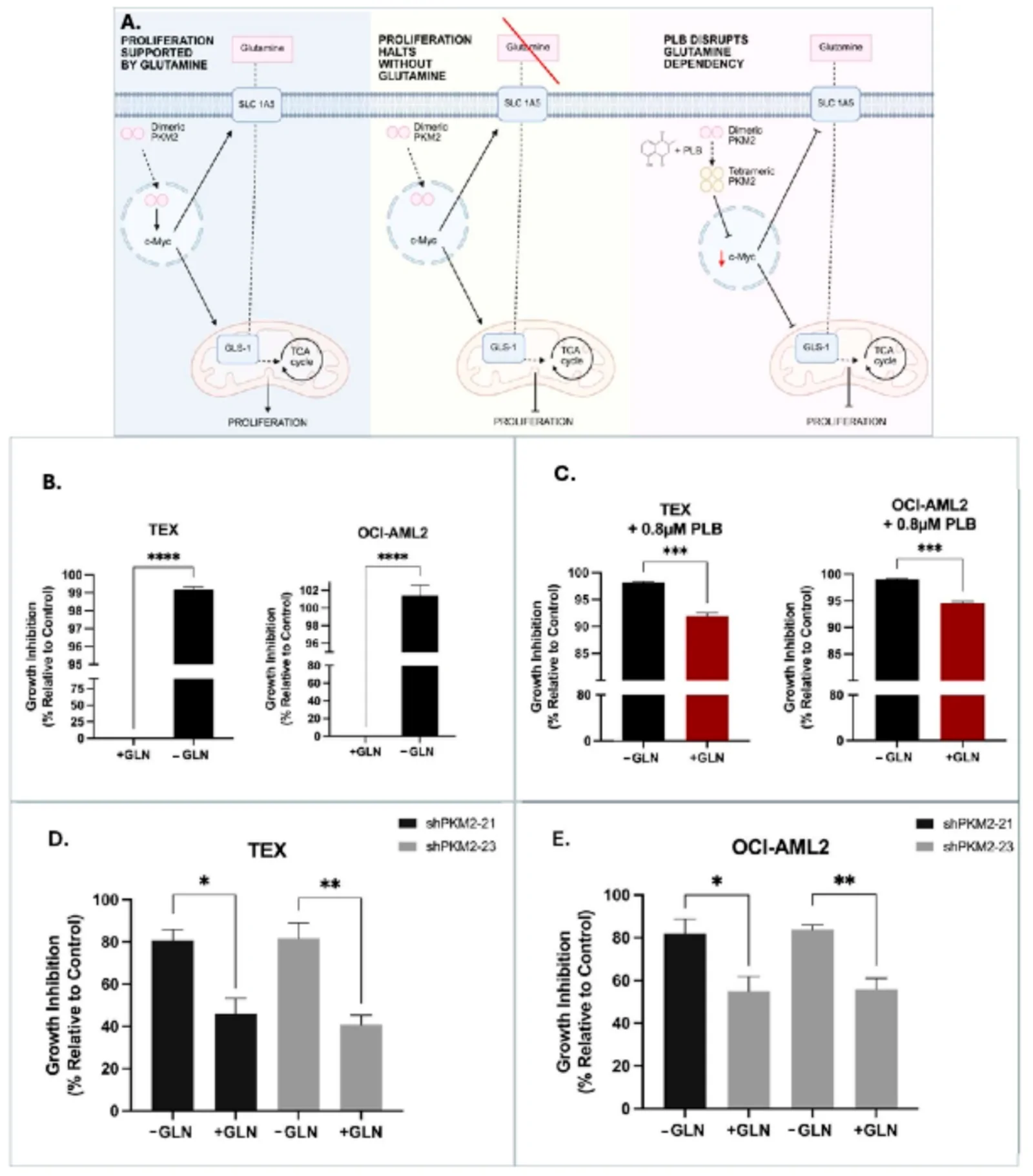
PKM2 modulation perturbates glutamine metabolism. (**A**) Schematic diagram depicting the proposed perturbations in glutamine metabolism under different scenarios. (**Left panel**): Under normal conditions, pyruvate kinase isoform M2 (PKM2)-induced c-Myc expression upregulates glutamine transporter (SLC1A5) and glutaminase 1 (GLS-1) expression to fuel glutamine uptake and catabolism, contributing to increased proliferation. (**Middle panel**): Glutamine withdrawal reduces proliferation. (**Right panel**): Plumbagin (PLB) treatment impairs PKM2 nuclear activity and c-Myc expression, reducing glutamine transporter expression, GLS-1 activity, and proliferation. Biorender, + N. Vrdoljak (2025) + http://Biorender.com. (**B**) TEX and OCI-AML2 cell growth inhibition in glutamine-free conditions normalized to cells cultured in normal conditions. (**C**) Growth inhibition in PLB-treated TEX and OCI-AML2 cells in normal and glutamine-free conditions, normalized to an untreated control cultured in normal conditions. (**D**) Growth inhibition in TEX and **(E)** OCI-AML2 PKM2 knockdown (shPKM2) cell lines in normal and glutamine-free conditions, normalized to an untreated TRC control cultured in normal conditions. * *p* ≤ 0.05, ** *p* ≤ 0.01, *** *p* ≤ 0.001, **** *p* ≤ 0.0001. Unpaired, two-tailed Student’s *t*-test. All data is represented as the mean ± standard error of mean. TCA: tricarboxylic acid; Gln: glutamine. (**B**–**D**), n = 3 biological replicates.

AML cells are dependent on glutamine and therefore overexpress transporters such as SLC1A5 and catabolic enzymes such as GLS-1 to support glutamine uptake and catabolism, respectively [4,16,43]. Figure 2A is a schematic overview highlighting downstream effects of PKM2 and the hypothesized consequences of metabolic perturbations on glutamine uptake and metabolism.

**Figure A1:**
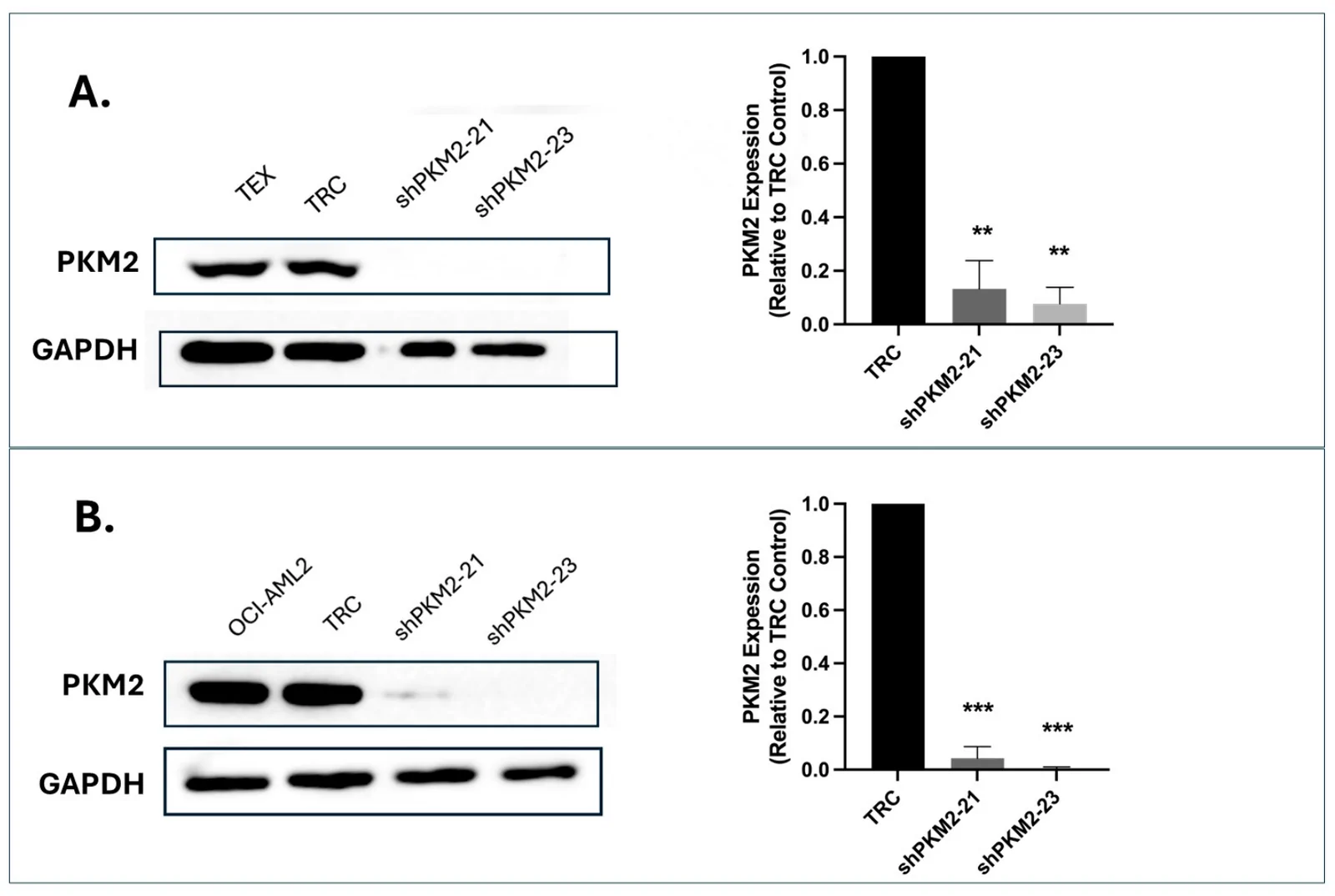
Knockdown of PKM2 reduces expression in vitro. Immunoblots and densitometry analysis of pyruvate kinase isoform M2 (PKM2) in PKM2 knockdown (shPKM2) constructs confirming genetic knockdown of PKM2 in (**A**) TEX and (**B**) OCI-AML2 cells. All shPKM2 models are normalized to TRC controls. ** *p* ≤ 0.01, *** *p* ≤ 0.001,. One-way ANOVA with Tukey’s post hoc test. All data is represented as the mean ± standard error of mean.

To demonstrate reliance on glutamine, leukemia cell lines were cultured in the presence or absence of glutamine. Comparing the effects of normal (i.e., glutamine-rich) and glutamine-free conditions, the absence of glutamine significantly inhibited AML cell growth (Figure 2B; *p* ≤ 0.0001). Interestingly, glutamine supplementation partially rescued cell growth under PLB treatment; the partial offset, as opposed to complete reversion of the inhibitory effect, indicating that PLB treatment affects glutamine metabolism (Figure 2C; *p* ≤ 0.001). Similar outcomes were observed in AML cell lines with reduced PKM2 expression, hereafter referred to as shPKM2 cells (Figure 2D,E and Figure A1; *p* ≤ 0.05).

These findings suggest a link between PLB, PKM2 modulation, and glutamine metabolism. Our previous findings suggest a link between PKM2 modulation and effects on downstream signaling pathways; however, its influence on glutamine metabolism has been unexplored. c-Myc is a key transcriptional regulator of glutamine metabolism, promoting expression of proteins such as SLC1A5 and GLS-1 [17,18,19]. As such, we hypothesized that PKM2 modulation may impair glutamine metabolism through the c-Myc/PKM2 axis. The partial restoration of proliferation following glutamine supplementation supports the role of glutamine-dependent processes in the anti-proliferative effects of PKM2 modulation; however, it also suggests that additional mechanisms are involved.

### 3.3. Plumbagin Reduces GLS-1 Expression

GLS-1 protein levels are elevated in head and neck squamous cell carcinoma, glioblastoma, lymphoma, and colorectal carcinoma [19,44,45,46]. In addition, an AML patient dataset from the Cancer Genome Atlas reports that glutaminase (GLS) expression is in the top 4% of all genes [16]. GLS-1 overexpression promotes glutamine catabolism through the production of glutamate and eventual sustainment of anapleurosis, enabling AML cell growth [16].

**Figure 3:**
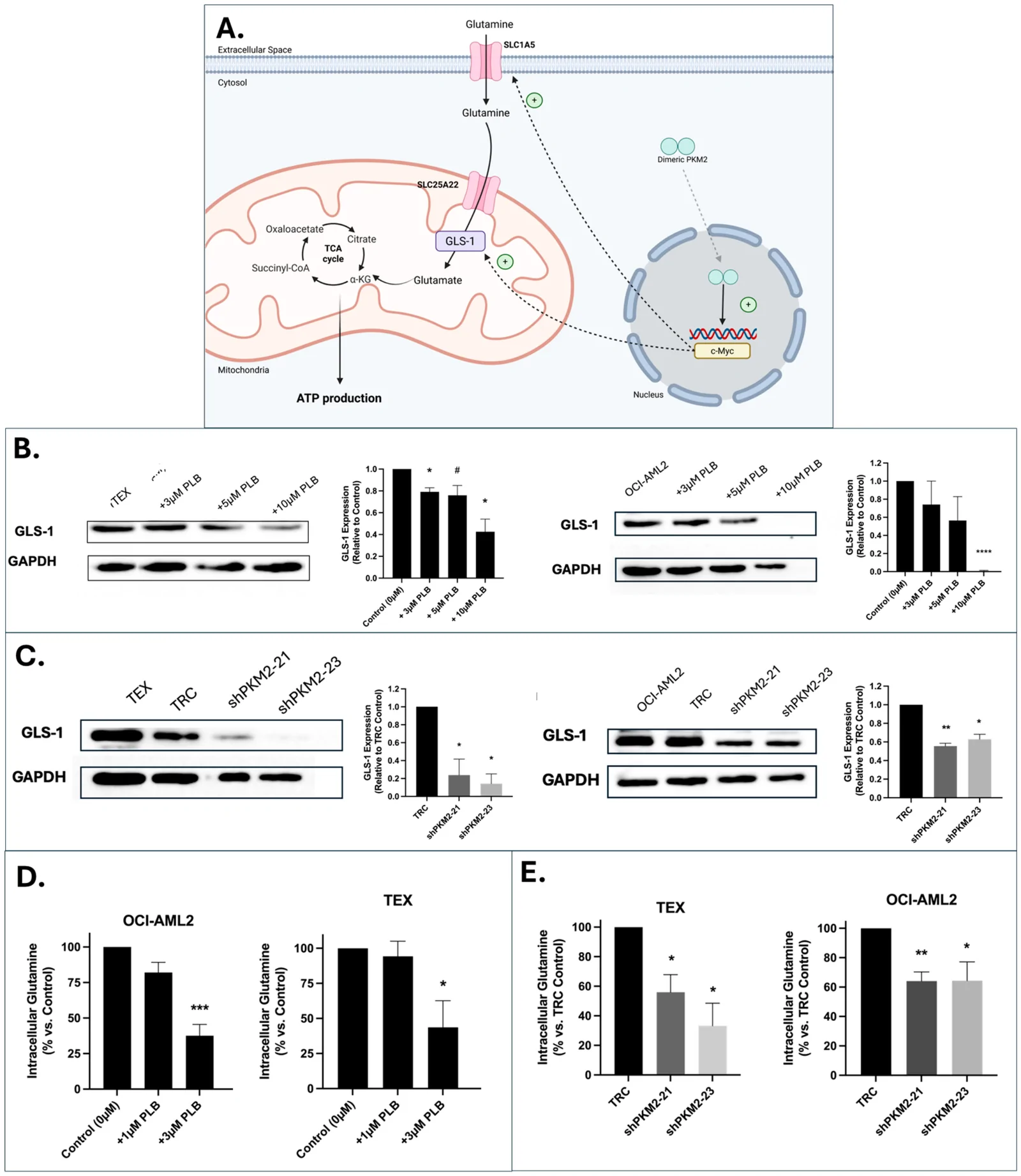
PKM2 modulation affects glutamine catabolism and uptake. (**A**) Schematic depiction of the effect of PKM2-mediated c-Myc expression on glutamine metabolism. c-Myc upregulates glutamine transporter (SLC1A5, SLC25A22) expression, increasing intracellular glutamine uptake, and upregulates glutaminase 1 (GLS-1) expression, increasing glutamate production, to propagate the tricarboxylic acid (TCA) cycle via glutamate-derived α-ketoglutarate (α-KG). Biorender, + N. Vrdoljak (2025) + http://Biorender.com. (**B**) Immunoblots and densitometry analysis of GLS-1/glutaminase C (GAC) expression in TEX and OCI-AML2 cell lines following PLB treatment. (**C**) Immunoblots and densitometry analysis of GLS-1/GAC expression in TEX and OCI-AML2 shPKM2 cell lines. (**D**) Intracellular glutamine concentrations in TEX and OCI-AML2 cell lines following plumbagin (PLB) treatment normalized to an untreated control. (**E**) Intracellular glutamine concentrations in TEX and OCI-AML2 PKM2 knockdown (shPKM2) cell lines normalized to a TRC control. # *p* = 0.05, * *p* ≤ 0.05, ** *p* ≤ 0.01, *** *p* ≤ 0.001, **** *p* ≤ 0.0001. Unpaired, two-tailed Student’s *t*-test (**B**,**C**,**E**). One-way ANOVA with Tukey’s post hoc test (**D**). All data is represented as the mean ± standard error of mean. ATP: adenose triphosphate. (**B**,**C**), n = 2; (**D**,**E**), n = 3.

Because of the role that c-Myc plays in regulating GLS-1 expression, along with previous work demonstrating that PKM2 modulation reduces c-Myc expression, we hypothesized that genetic or pharmacological modulation of PKM2 would result in suppressed GLS-1 expression (Figure 3A). Indeed, immunoblotting revealed that AML cells treated with PLB demonstrate a significant and dose-dependent decrease in GLS-1 protein levels (Figure 3B; TEX *p* ≤ 0.05, OCI-AML2 *p* ≤ 0.0001). Similarly, endogenous levels of GLS-1 were reduced in shPKM2 cells compared to TRC controls, further supporting a relationship between PKM2 modulation and GLS-1 expression. (Figure 3C; *p* ≤ 0.05).

### 3.4. Plumbagin Reduces Glutamine Uptake into Cell

c-Myc binds directly to an SLC1A5 promoter region, consequently upregulating transporter expression and glutamine uptake [18]. Therefore, we hypothesized that PKM2-mediated c-Myc suppression would impact glutamine uptake. The results demonstrate that PLB treatment significantly decreases intracellular glutamine concentrations in both TEX and OCI-AML2 cell lines (Figure 3D; TEX F(3,6) = 36.71, *p* ≤ 0.001; OCI-AML2 F(3,7) = 11.84, *p* ≤ 0.01). Similarly, endogenous glutamine concentrations were reduced in shPKM2 cells compared to TRC cell lines (Figure 3E; TEX F(2,3) = 15.20, *p* ≤ 0.05; OCI-AML2 F(2,3) = 45.76, *p* ≤ 0.01). These findings are consistent with the hypothesis of the c-Myc/PKM2 axis impacting glutamine metabolism, suggesting that PKM2 modulation is associated with impaired glutamine uptake in AML cell lines.

## 4. Discussion

A hallmark of AML is metabolic reprogramming, which enables cells to become flexible in terms of nutrient utilization to maintain rapid proliferation. Deregulated metabolism in malignant cells increases glucose uptake yet reduces glycolytic flux, leading to an accumulation of upstream glycolytic intermediates that are ultimately diverted to anabolic pathways [5]. As such, AML cells utilize alternative fuel sources such as fatty acids and glutamine to sustain mitochondrial respiration through anapleurosis. The vast differences in metabolism in malignancy have isolated metabolic targeting as an attractive approach for AML drug therapy [11,12,47].

Given the role of glutamine metabolism in AML, pharmacological targeting of this pathway has been investigated. Mechanistic studies observe that genetic suppression of SLC1A5 disrupts glutamine uptake and leads to impaired growth of AML cells in vitro and in vivo [14,43]. Pharmacological inhibition of SLC1A5-mediated glutamine uptake via l-γ-Glutamyl-p-nitroanilide (GPNA) has been investigated, and V-9302, a small-molecule inhibitor with broad metabolic impacts that was originally investigated as an inhibitor of SLC1A5, suppresses in vivo tumor growth by disrupting glutamine uptake [14,15]. While SLC1A5 imports glutamine, GLS-1 is the mitochondrial enzyme initiating glutamine catabolism [48]. GLS-1 deaminates glutamine to create glutamate, which is subsequently converted to α-ketoglutarate to fuel mitochondrial respiration. Genetic knockdown of GLS-1 suppresses AML cell growth and survival by impairing glutamine metabolism, and pharmacological inhibitors such as CB-839 have demonstrated efficacy in inhibiting AML growth in cell lines and patient samples [16,49,50]. Regardless, there are no clinically available inhibitors of glutamine transport or catabolism.

The utilization of glutamine is tightly regulated by oncogenic signaling pathways including c-Myc, which promote the overexpression of glutamine transporters and enzymes associated with catabolism; the c-Myc pathway is partially controlled by a positive feedback loop with PKM2 [17,18,19]. PLB is an identified activator of PKM2 and has been reported as cytotoxic towards many types of cancers, including AML [35,36,37,38,39,40,41,51,52,53,54,55]. Our previous work demonstrated that PLB-induced activation of PKM2 disrupts the native oligomeric state, reducing nuclear translocation and suppressing c-Myc expression [34]. Building on these observations, we sought to investigate whether PKM2 modulation influences glutamine metabolism as a downstream consequence of c-Myc suppression.

Although PKM2 activation is an important mechanism promoting PLB-mediated anti-AML activity, PLB has been previously reported to influence redox status, promote apoptosis and affect signaling pathways [35,36,56]. For example, PLB has been observed to disrupt downstream β-catenin and block STAT3 phosphorylation and activation [40,41]. Therefore, while the c-Myc/PKM2 axis provides a framework for understanding the metabolic effects observed in this study, alternative pathways likely also contribute to PLB-mediated AML cytotoxicity.

Despite these pleiotropic effects, our previous findings identifying PLB as a PKM2 activator provide an opportunity to further investigate the downstream metabolic consequences of PKM2 modulation. PLB-mediated PKM2 activation reduced intracellular glutamine uptake and GLS-1 expression, supporting the role of PKM2 in regulating cell metabolism beyond its canonical role in glycolysis. Supplementation of glutamine in PLB-treated cells partially offsets inhibitory effects, suggesting that while pharmacological modulation of PKM2 may dampen the response to glutamine, it does not completely eliminate a response; this points to mechanisms beyond glutamine metabolism that do not allow for a full response. Nevertheless, the partial response supports the hypothesis that PKM2 modulation influences glutamine uptake and catabolism. In addition, these results also provide insight into the potential for synergy with drugs targeting glutamine uptake and metabolism. Cells with reduced PKM2 expression and activity experience a similar trend, pointing to PKM2 activity as a central regulator of glutamine metabolism through its regulation of c-Myc. Differing from other pharmaceutical agents that directly inhibit glutamine utilization through suppression of glutamine transporters or glutaminolysis enzymes specifically, PLB induces a change in glutamine metabolism indirectly through the c-Myc/PKM2 axis. Targeting PKM2 as an upstream regulator has the potential to influence multiple metabolic pathways simultaneously, as opposed to inhibition of a single protein.

To further characterize the metabolic consequences of PLB-induced PKM2 activation, future studies focusing on glutamine transporters and other catabolic enzymes regulated by c-Myc should be conducted. While the rate of intracellular glutamine uptake was evaluated, transcriptional analysis of transporters including SLC1A5 with quantitative reverse-transcription polymerase chain reaction (qRT-PCR) would provide a direct evaluation of transcriptional inhibition due to PLB treatment. Conversely, while expression of GLS-1 was assessed via immunoblotting, enzyme activity was not measured; further investigation utilizing activity assays would provide additional insight into the functionality of the protein, and utilization of glutamine within the cell. Finally, conducting metabolomic assessments, including stable isotope tracing experiments using labeled glutamine substrates, would provide a comprehensive overview of the metabolic pathways that are affected by PLB-induced PKM2 activation. Isotope tracing metabolomics would allow for direct evaluation of glutamine carbon utilization through different pathways such as glutaminolysis and the tricarboxylic acid cycle, potentially identifying other metabolic vulnerabilities induced by PLB-induced PKM2 activation. Together, these strategies would further define the metabolic influences following PKM2 modulation and position its therapeutic potential in AML.

## 5. Conclusions

Altogether, the results of this study reinforce the role of c-Myc-mediated regulation of glutamine utilization in AML and expand the regulatory axis upstream to include PKM2 activity as a modulator of these changes. These data expand previous findings that identified c-Myc inhibition as a converging cellular consequence of PKM2 activity regulation, recognizing the additional implications of PKM2 modulation beyond glycolytic metabolism and nuclear function [34]. This distinguishes PKM2 as a regulator of central cell metabolism and highlights the use of PKM2-targeting as a promising strategy in impairing glutamine-dependent metabolism in AML.

## Figures and Tables

**Table 2:** Immunoblotting antibodies.

Antibody	Isotype	Molecular Weight (kDa)	Supplier
Human GLS-1	Rabbit	55–65	56750; Cell Signaling Technology
Human GAPDH	Rabbit	37	AB210504; Abcam
Human PKM2	Rabbit	60	4053; Cell Signaling Technology

## Data Availability

Data supporting the findings of this study are available from the corresponding author upon reasonable request.

## Abbreviations

The following abbreviations are used in this manuscript:

AML	Acute myeloid leukemia
AG	Aerobic glycolysis
BCA	Bicinchoninic acid
EDTA	Ethylenediaminetetraacetic acid
FBS	Fetal bovine serum
GLS-1	Glutaminase 1
GPNA	L-γ-Glutamyl-p-nitroanilide
HRP	Horseradish peroxidase
IMDM	Iscove’s Modified Dulbecco’s Medium
MES	2-(N-morpholino)ethanesulfonic acid
OCI-AML2	Ontario Cancer Institute-acute myeloid leukemia-2
OX PHOS	Oxidative phosphorylation
PAGE	Polyacrylamide gel electrophoresis
PBMCs	Peripheral blood mononuclear cells
PBS	Phosphate-buffered saline
PIC	Protease inhibitor cocktail
PK	Pyruvate kinase
PKM1	Pyruvate kinase isoform M1
PKM2	Pyruvate kinase isoform M2
PLB	Plumbagin
PVDF	Polyvinylidene difluoride
RIPA	Radioimmunoprecipitation buffer
SDS	Sodium dodecyl sulfate
SLC-1A5	Solute carrier family 1 member 5
shPKM2	shRNA specific for PKM2
shRNA	Short hairpin ribonucleic acid
TBS-T	Tris-buffered saline with Tween20
qRT-PCR	Quantitative reverse-transcription polymerase chain reaction

## References

[1] Döhner H., Weisdorf D.J., Bloomfield C.D. (2015). Acute Myeloid Leukemia. N. Engl. J. Med..

[2] Roma A., Tcheng M., Ahmed N., Walker S., Jayanth P., Minden M.D., Reisz J.A., D’Alessandro A., Rohlena J., Spagnuolo P.A. (2022). Shikonin Impairs Mitochondrial Activity to Selectively Target Leukemia Cells. Phytomed. Plus.

[3] Tcheng M., Roma A., Ahmed N., Smith R.W., Jayanth P., Minden M.D., Schimmer A.D., Hess D.A., Hope K., Rea K.A. (2021). Very Long Chain Fatty Acid Metabolism Is Required in Acute Myeloid Leukemia. Blood.

[4] Chen W.-L., Wang J.-H., Zhao A.-H., Xu X., Wang Y.-H., Chen T.-L., Li J.-M., Mi J.-Q., Zhu Y.-M., Liu Y.-F. (2014). A Distinct Glucose Metabolism Signature of Acute Myeloid Leukemia with Prognostic Value. Blood.

[5] Vander Heiden M.G., Cantley L.C., Thompson C.B. (2009). Understanding the Warburg Effect: The Metabolic Requirements of Cell Proliferation. Science.

[6] Wang Y.-H., Scadden D.T. (2015). Targeting the Warburg Effect for Leukemia Therapy: Magnitude Matters. Mol. Cell. Oncol..

[7] Warburg O. (1956). On the Origin of Cancer Cells. Science.

[8] Chen Y., Xu Q., Ji D., Wei Y., Chen H., Li T., Wan B., Yuan L., Huang R., Chen G. (2016). Inhibition of Pentose Phosphate Pathway Suppresses Acute Myelogenous Leukemia. Tumor Biol..

[9] Bhanot H., Weisberg E.L., Reddy M.M., Nonami A., Neuberg D., Stone R.M., Podar K., Salgia R., Griffin J.D., Sattler M. (2017). Acute Myeloid Leukemia Cells Require 6-Phosphogluconate Dehydrogenase for Cell Growth and NADPH-Dependent Metabolic Reprogramming. Oncotarget.

[10] Zhong J., Huang K., Xie S., Tan A., Peng J., Nie D., Ma L., Li Y. (2024). *PHGDH* Is Key to a Prognostic Multigene Signature and a Potential Therapeutic Target in Acute Myeloid Leukemia. J. Cancer.

[11] Mishra S.K., Millman S.E., Zhang L. (2023). Metabolism in Acute Myeloid Leukemia: Mechanistic Insights and Therapeutic Targets. Blood.

[12] Xiao Y., Hu B., Guo Y., Zhang D., Zhao Y., Chen Y., Li N., Yu L. (2023). Targeting Glutamine Metabolism as an Attractive Therapeutic Strategy for Acute Myeloid Leukemia. Curr. Treat. Options Oncol..

[13] Wang D., Tan G., Wang H., Chen P., Hao J., Wang Y. (2019). Identification of Novel Serum Biomarker for the Detection of Acute Myeloid Leukemia Based on Liquid Chromatography-Mass Spectrometry. J. Pharm. Biomed. Anal..

[14] Ni F., Yu W.-M., Li Z., Graham D.K., Jin L., Kang S., Rossi M.R., Li S., Broxmeyer H.E., Qu C.-K. (2019). Critical Role of ASCT2-Mediated Amino Acid Metabolism in Promoting Leukaemia Development and Progression. Nat. Metab..

[15] Li Q.-Q., Pan S.-Y., Chen Q.-Y., Zhou W., Wang S.-Q. (2021). Effect of Competitive Antagonist of Transmembrane Glutamine Flux V-9302 on Apoptosis of Acute Myeloid Leukemia Cell Lines HL-60 and KG-1. Zhongguo Shi Yan Xue Ye Xue Za Zhi.

[16] Matre P., Velez J., Jacamo R., Qi Y., Su X., Cai T., Chan S.M., Lodi A., Sweeney S.R., Ma H. (2016). Inhibiting Glutaminase in Acute Myeloid Leukemia: Metabolic Dependency of Selected AML Subtypes. Oncotarget.

[17] Amaya M.L., Inguva A., Pei S., Jones C., Krug A., Ye H., Minhajuddin M., Winters A., Furtek S.L., Gamboni F. (2022). The STAT3-MYC Axis Promotes Survival of Leukemia Stem Cells by Regulating SLC1A5 and Oxidative Phosphorylation. Blood.

[18] Zhao X., Petrashen A.P., Sanders J.A., Peterson A.L., Sedivy J.M. (2019). SLC1A5 Glutamine Transporter Is a Target of MYC and Mediates Reduced mTORC1 Signaling and Increased Fatty Acid Oxidation in Long-lived *Myc* Hypomorphic Mice. Aging Cell.

[19] Gao P., Tchernyshyov I., Chang T.-C., Lee Y.-S., Kita K., Ochi T., Zeller K.I., De Marzo A.M., Van Eyk J.E., Mendell J.T. (2009). C-Myc Suppression of miR-23a/b Enhances Mitochondrial Glutaminase Expression and Glutamine Metabolism. Nature.

[20] David C.J., Chen M., Assanah M., Canoll P., Manley J.L. (2010). HnRNP Proteins Controlled by C-Myc Deregulate Pyruvate Kinase mRNA Splicing in Cancer. Nature.

[21] Jha R.K., Kouzine F., Levens D. (2023). MYC Function and Regulation in Physiological Perspective. Front. Cell Dev. Biol..

[22] Zahra K., Dey T., Ashish, Mishra S.P., Pandey U. (2020). Pyruvate Kinase M2 and Cancer: The Role of PKM2 in Promoting Tumorigenesis. Front. Oncol..

[23] Anastasiou D., Yu Y., Israelsen W.J., Jiang J.-K., Boxer M.B., Hong B.S., Tempel W., Dimov S., Shen M., Jha A. (2012). Pyruvate Kinase M2 Activators Promote Tetramer Formation and Suppress Tumorigenesis. Nat. Chem. Biol..

[24] Mazurek S. (2011). Pyruvate Kinase Type M2: A Key Regulator of the Metabolic Budget System in Tumor Cells. Int. J. Biochem. Cell Biol..

[25] Yang W., Xia Y., Ji H., Zheng Y., Liang J., Huang W., Gao X., Aldape K., Lu Z. (2011). Nuclear PKM2 Regulates β-Catenin Transactivation upon EGFR Activation. Nature.

[26] Yang W., Zheng Y., Xia Y., Ji H., Chen X., Guo F., Lyssiotis C.A., Aldape K., Cantley L.C., Lu Z. (2012). ERK1/2-Dependent Phosphorylation and Nuclear Translocation of PKM2 Promotes the Warburg Effect. Nat. Cell Biol..

[27] Gao X., Wang H., Yang J.J., Liu X., Liu Z.-R. (2012). Pyruvate Kinase M2 Regulates Gene Transcription by Acting as a Protein Kinase. Mol. Cell.

[28] Vander Heiden M.G., Christofk H.R., Schuman E., Subtelny A.O., Sharfi H., Harlow E.E., Xian J., Cantley L.C. (2010). Identification of Small Molecule Inhibitors of Pyruvate Kinase M2. Biochem. Pharmacol..

[29] Ning X., Qi H., Li R., Li Y., Jin Y., McNutt M.A., Liu J., Yin Y. (2017). Discovery of Novel Naphthoquinone Derivatives as Inhibitors of the Tumor Cell Specific M2 Isoform of Pyruvate Kinase. Eur. J. Med. Chem..

[30] Li J., Li S., Guo J., Li Q., Long J., Ma C., Ding Y., Yan C., Li L., Wu Z. (2018). Natural Product Micheliolide (MCL) Irreversibly Activates Pyruvate Kinase M2 and Suppresses Leukemia. J. Med. Chem..

[31] Jiang J., Walsh M.J., Brimacombe K.R., Anastasiou D., Yu Y., Israelsen W.J., Hong B.-S., Tempel W., Dimov S., Veith H. (2010). ML265: A Potent PKM2 Activator Induces Tetramerization and Reduces Tumor Formation and Size in a Mouse Xenograft Model. Probe Reports from the NIH Molecular Libraries Program.

[32] Tang X., Ding Q., Chen C., Chen F., Zhou X., Hong C.J., Pan W. (2019). Micheliolide Inhibits Gastric Cancer Growth in Vitro and in Vivo via Blockade of the IL-6/STAT3 Pathway. Pharmazie.

[33] Petrocelli G., Marrazzo P., Bonsi L., Facchin F., Alviano F., Canaider S. (2023). Plumbagin, a Natural Compound with Several Biological Effects and Anti-Inflammatory Properties. Life.

[34] Vrdoljak N., Parfenova E.N., Mosca D.A., Roma A., Minden M.D., Spagnuolo P.A. (2026). PKM2 Modulation Regulates Leukemia Cell Survival. Cancer Metab..

[35] Xu K.-H., Lu D.-P. (2010). Plumbagin Induces ROS-Mediated Apoptosis in Human Promyelocytic Leukemia Cells in Vivo. Leuk. Res..

[36] Fu C., Gong Y., Shi X., Sun Z., Niu M., Sang W., Xu L., Zhu F., Wang Y., Xu K. (2016). Plumbagin Reduces Chronic Lymphocytic Leukemia Cell Survival by Downregulation of Bcl-2 but Upregulation of the Bax Protein Level. Oncol. Rep..

[37] Sun J., McKallip R.J. (2011). Plumbagin Treatment Leads to Apoptosis in Human K562 Leukemia Cells through Increased ROS and Elevated TRAIL Receptor Expression. Leuk. Res..

[38] Gaascht F., Teiten M.-H., Cerella C., Dicato M., Bagrel D., Diederich M. (2014). Plumbagin Modulates Leukemia Cell Redox Status. Molecules.

[39] Cao Y., Yin X., Jia Y., Liu B., Wu S., Shang M. (2018). Plumbagin, a Natural Naphthoquinone, Inhibits the Growth of Esophageal Squamous Cell Carcinoma Cells through Inactivation of STAT3. Int. J. Mol. Med..

[40] Hafeez B.B., Jamal M.S., Fischer J.W., Mustafa A., Verma A.K. (2012). Plumbagin, a Plant Derived Natural Agent Inhibits the Growth of Pancreatic Cancer Cells in in Vitro and in Vivo via Targeting EGFR, Stat3 and NF-κB Signaling Pathways. Int. J. Cancer.

[41] Sakunrangsit N., Ketchart W. (2019). Plumbagin Inhibits Cancer Stem-like Cells, Angiogenesis and Suppresses Cell Proliferation and Invasion by Targeting Wnt/β-Catenin Pathway in Endocrine Resistant Breast Cancer. Pharmacol. Res..

[42] Rodrigo R., Mendis N., Ibrahim M., Ma C., Kreinin E., Roma A., Berg S., Blay J., Spagnuolo P.A. (2019). Knockdown of BNIP3L or SQSTM1 Alters Cellular Response to Mitochondria Target Drugs. Autophagy.

[43] Willems L., Jacque N., Jacquel A., Neveux N., Trovati Maciel T., Lambert M., Schmitt A., Poulain L., Green A.S., Uzunov M. (2013). Inhibiting Glutamine Uptake Represents an Attractive New Strategy for Treating Acute Myeloid Leukemia. Blood.

[44] Kim J.H., Lee K.J., Seo Y., Kwon J.-H., Yoon J.P., Kang J.Y., Lee H.J., Park S.J., Hong S.P., Cheon J.H. (2018). Effects of Metformin on Colorectal Cancer Stem Cells Depend on Alterations in Glutamine Metabolism. Sci. Rep..

[45] Wise D.R., DeBerardinis R.J., Mancuso A., Sayed N., Zhang X.-Y., Pfeiffer H.K., Nissim I., Daikhin E., Yudkoff M., McMahon S.B. (2008). Myc Regulates a Transcriptional Program That Stimulates Mitochondrial Glutaminolysis and Leads to Glutamine Addiction. Proc. Natl. Acad. Sci. USA.

[46] Yang J., Chen F., Lang L., Yang F., Fu Z., Martinez J., Cho A., Saba N.F., Teng Y. (2024). Therapeutic Targeting of the GLS1–c-Myc Positive Feedback Loop Suppresses Glutaminolysis and Inhibits Progression of Head and Neck Cancer. Cancer Res..

[47] Hensley C.T., Wasti A.T., DeBerardinis R.J. (2013). Glutamine and Cancer: Cell Biology, Physiology, and Clinical Opportunities. J. Clin. Investig..

[48] Cluntun A.A., Lukey M.J., Cerione R.A., Locasale J.W. (2017). Glutamine Metabolism in Cancer: Understanding the Heterogeneity. Trends Cancer.

[49] Zacharias N.M., Baran N., Shanmugavelandy S.S., Lee J., Lujan J.V., Dutta P., Millward S.W., Cai T., Wood C.G., Piwnica-Worms D. (2019). Assessing Metabolic Intervention with a Glutaminase Inhibitor in Real-Time by Hyperpolarized Magnetic Resonance in Acute Myeloid Leukemia. Mol. Cancer Ther..

[50] Jacque N., Ronchetti A.M., Larrue C., Meunier G., Birsen R., Willems L., Saland E., Decroocq J., Maciel T.T., Lambert M. (2015). Targeting Glutaminolysis Has Antileukemic Activity in Acute Myeloid Leukemia and Synergizes with BCL-2 Inhibition. Blood.

[51] Sandur S.K., Ichikawa H., Sethi G., Ahn K.S., Aggarwal B.B. (2006). Plumbagin (5-Hydroxy-2-Methyl-1,4-Naphthoquinone) Suppresses NF-κB Activation and NF-κB-Regulated Gene Products Through Modulation of P65 and IκBα Kinase Activation, Leading to Potentiation of Apoptosis Induced by Cytokine and Chemotherapeutic Agents. J. Biol. Chem..

[52] Zhang X.Q., Yang C.Y., Rao X.F., Xiong J.P. (2016). Plumbagin Shows Anti-Cancer Activity in Human Breast Cancer Cells by the Upregulation of P53 and P21 and Suppression of G1 Cell Cycle Regulators. Eur. J. Gynaecol. Oncol..

[53] Hafeez B.B., Fischer J.W., Singh A., Zhong W., Mustafa A., Meske L., Sheikhani M.O., Verma A.K. (2015). Plumbagin Inhibits Prostate Carcinogenesis in Intact and Castrated PTEN Knockout Mice via Targeting PKCϵ, Stat3, and Epithelial-to-Mesenchymal Transition Markers. Cancer Prev. Res..

[54] Raghu D., Karunagaran D. (2014). Plumbagin Downregulates Wnt Signaling Independent of P53 in Human Colorectal Cancer Cells. J. Nat. Prod..

[55] Chen C.-A., Chang H.-H., Kao C.-Y., Tsai T.-H., Chen Y.-J. (2009). Plumbagin, Isolated from Plumbago Zeylanica, Induces Cell Death through Apoptosis in Human Pancreatic Cancer Cells. Pancreatology.

[56] Wu H., Dai X., Wang E. (2016). Plumbagin Inhibits Cell Proliferation and Promotes Apoptosis in Multiple Myeloma Cells through Inhibition of the PI3K/Akt-mTOR Pathway. Oncol. Lett..

